# Biallelic loss-of-function in *NRAP* is a cause of recessive dilated cardiomyopathy

**DOI:** 10.1371/journal.pone.0245681

**Published:** 2021-02-03

**Authors:** Juha W. Koskenvuo, Inka Saarinen, Saija Ahonen, Johanna Tommiska, Sini Weckström, Eija H. Seppälä, Sari Tuupanen, Tiia Kangas-Kontio, Jennifer Schleit, Krista Heliö, Julie Hathaway, Anders Gummesson, Pia Dahlberg, Tiina H. Ojala, Ville Vepsäläinen, Ville Kytölä, Mikko Muona, Johanna Sistonen, Pertteli Salmenperä, Massimiliano Gentile, Jussi Paananen, Samuel Myllykangas, Tero-Pekka Alastalo, Tiina Heliö

**Affiliations:** 1 Blueprint Genetics, a Quest Diagnostics Company, Espoo, Finland; 2 Heart and Lung Center, Helsinki University Hospital, University of Helsinki, Helsinki, Finland; 3 Blueprint Genetics Inc, a Quest Diagnostics Company, Seattle, Washington, United States of America; 4 Department of Clinical Genetics and Genomics, Sahlgrenska University Hospital, Gothenburg, Sweden; 5 Department of Cardiology, Sahlgrenska University Hospital, Gothenburg, Sweden; 6 Department of Pediatric Cardiology, Helsinki University Hospital and University of Helsinki, Helsinki, Finland; 7 Heart Center, Kuopio University Hospital, Kuopio, Finland; Ohio State University Wexner Medical Center, UNITED STATES

## Abstract

**Background:**

Familial dilated cardiomyopathy (DCM) is typically a monogenic disorder with dominant inheritance. Although over 40 genes have been linked to DCM, more than half of the patients undergoing comprehensive genetic testing are left without molecular diagnosis. Recently, biallelic protein-truncating variants (PTVs) in the nebulin-related anchoring protein gene (*NRAP*) were identified in a few patients with sporadic DCM.

**Methods and results:**

We determined the frequency of rare *NRAP* variants in a cohort of DCM patients and control patients to further evaluate role of this gene in cardiomyopathies. A retrospective analysis of our internal variant database consisting of 31,639 individuals who underwent genetic testing (either panel or direct exome sequencing) was performed. The DCM group included 577 patients with either a confirmed or suspected DCM diagnosis. A control cohort of 31,062 individuals, including 25,912 individuals with non-cardiac (control group) and 5,150 with non-DCM cardiac indications (Non-DCM cardiac group). Biallelic (n = 6) or two (n = 5) *NRAP* variants (two PTVs or PTV+missense) were identified in 11 unrelated probands with DCM (1.9%) but none of the controls. None of the 11 probands had an alternative molecular diagnosis. Family member testing supports co-segregation. Biallelic or potentially biallelic *NRAP* variants were enriched in DCM vs. controls (OR 1052, p<0.0001). Based on the frequency of *NRAP* PTVs in the gnomAD reference population, and predicting full penetrance, biallelic *NRAP* variants could explain 0.25%-2.46% of all DCM cases.

**Conclusion:**

Loss-of-function in *NRAP* is a cause for autosomal recessive dilated cardiomyopathy, supporting its inclusion in comprehensive genetic testing.

## Introduction

Dilated cardiomyopathy (DCM) is characterized by left ventricular enlargement and systolic dysfunction in the absence of other etiological causes [1]. It is typically an adult-onset disease but disease onset may take place as early as in infancy. Genetic DCM has incomplete, age-dependent penetrance and presentation may vary even within the same family ranging from asymptomatic to end-stage heart failure and sudden cardiac death (SCD). The prevalence of DCM in the general population is estimated int the range of 1:500 to 1:3,000 [2–4].

Familial DCM is typically considered to be a monogenic disorder following most commonly an autosomal dominant pattern of inheritance [1,2,5]. However, X-linked, recessive and mitochondrial inheritance patterns have been observed [6]. As much as 30–50% of DCM is thought to be genetic or familial [6,7]. Over 40 genes encoding proteins of cytoskeleton, sarcomere, nuclear envelope, ion channels, and intercellular junction such as *TTN*, *LMNA*, *MYH7*, *FLNC*, *DSP*, *TNNT2*, *RBM20*, *DES*, *TPM1* and *DMD* contribute to the monogenic forms of DCM [7–11].

Recently, biallelic protein-truncating variants (PTVs) in the nebulin-related anchoring protein gene (*NRAP*) have been identified in a few patients with severe sporadic DCM [12–15], and have been proposed to cause low-penetrant recessive DCM (Table 1). However, two healthy individuals (age 33 and 35) in these families had the same homozygous PTV, which was considered to partially question the variants’ pathogenicity. *NRAP* is not yet officially a morbid OMIM gene and has not yet been curated by ClinGen (NIH) or Genomics England PanelApp [16]. Thus, it is absent from most commercially available gene panels at the moment.

**Table 1:** Previously reported patients with biallelic truncations in *NRAP*.

	Chromosomal position	Transcript; exon	Variant	GnomAD allele frequency	Pheno-type	Age at onset (years)gender	LVEDD (mm)	EF (%)	Other clinical features	References
1	10:115355414	NM_198060.3 Exon 38/42	c.4504 C>T, p.(Arg1502*)^#^	92/282804	DCM	26 M	71	15	Biventricular heart failure after prolonged viral-like illness, ventricular tachycardia, CK 68 U/l (normal: 39–308), NT-proBNP 5154 pg/ml, and TnT 15.04 ng/ml. 36- year old brother^#^ is healthy.	Truszkowska 2017 (12)
2	10:115413838–115413845	NM_001261463 Exon 5/42	c.400_407del,p.(Cys134Serfs*12)^#^	0/251404	DCM	NA	NA	NA	NA	Monies 2017 (13)
3	10:115400070	NM_001261463 Exon 14/42	c.1344T>A, p.(Tyr448)*^#^	34/263614	DCM	3.5 F	>3SD	15	DCM after mild respiratory viral infection, suspected myocarditis, LVEDV 245ml/m^2^. Died before planned Htx. In autopsy no signs of myocarditis.	Vasilescu 2018 (14)
4	10:115413838–115413845	NM_001261463 Exon 5/42	c.400_407del,p.(Cys134Serfs*12)^#^	0/251404	DCM	1 F		15	Cardiac arrest. CK 163 U/l (normal 26–168), Brother died at 17 months for cardiomyopathy, genotype unknown. Father^#^ is healthy	Ahmed 2019 (15)

Abbreviations: DCM, dilated cardiomyopathy; F, female; GnomAD; Allele frequency in gnomAD, Htx, heart transplantation; LVEDD; Left ventricular end-diastolic diameter; M, male; NA, not available; Pheno, phenotype

^#^, homozygous. No homozygotes for these variants are present in the gnomAD reference population cohort.

Since both enrichment and co-segregation of *NRAP* variants in DCM are unknown, our aims were to 1) evaluate whether patients who underwent genetic testing due to DCM have a higher frequency of *NRAP* variants compared to controls, 2) to study co-segregation of the *NRAP* variants, and 3) to define genotype-to-phenotype associations in *NRAP*-associated cardiomyopathy.

## Materials and methods

### Patients

The cohort represents 31,639 consecutive patients referred to genetic testing relying either on whole exome sequencing platform (WES; n = 24,630) or 4,600 gene high-quality next generation sequencing assay (HQSA; n = 7009) after January 2017. The inclusion criteria for DCM group (see later) was referral to genetic testing due to diagnosis or clinical suspicion of DCM.

This registry study complies with the Declaration of Helsinki. Patients who consented for Blueprint Genetics to contact them in relation to future research findings after initial testing, were contacted through their referring healthcare professional when possibly diagnostic biallelic variants in *NRAP* gene were found in sequence data. Patients living in the Helsinki University Hospital (HUS) region in Southern Finland were recruited to the Inherited Cardiomyopathies Study or KidCMP Study, and segregation studies were carried out when possible. Participants of the Inherited Cardiomyopathies study gave written informed consent, and the study was approved by the Ethical Review Committee of The Department of Medicine, University of Helsinki (Dnro 307/13/03/01/2011, HUS/3225/2018, TMK11§274,16.12.2015). This study has permission from Statistics Finland and Ministry of Social Affairs and Health to obtain clinical data from deceased patients for research purposes.

### Sequencing

Sample preparation including DNA isolation, fragmentation, library preparation techniques, bioinformatics, and quality control were similar for both WES and HQSA.

When required, the total genomic DNA was extracted from the biological sample using bead-based method. DNA quality and quantity were assessed using electrophoretic methods. After assessment of DNA quality, qualified genomic DNA sample was randomly fragmented using non-contact, isothermal sonochemistry processing. Sequencing library was prepared by ligating sequencing adapters to both ends of DNA fragments. Sequencing libraries were size-selected with bead-based method to ensure optimal template size and amplified by polymerase chain reaction. Regions of interest (exons and intronic targets) were targeted using hybridization-based target capture method. The quality of the completed sequencing library was controlled by ensuring the correct template size and quantity and to eliminate the presence of leftover primers and adapter-adapter dimers. Ready sequencing libraries that passed the quality control were sequenced using the Illumina's sequencing-by-synthesis method using paired-end sequencing (150 by 150 bases). Primary data analysis converting images into base calls and associated quality scores was carried out by the sequencing instrument using Illumina's proprietary software, generating CBCL files as the final output.

Base called raw sequencing data was transformed into FASTQ format using Illumina's software (bcl2fastq). Sequence reads of each sample were mapped to the human reference genome (GRCh37/hg19). Burrows-Wheeler Aligner (BWA-MEM) software was used for read alignment. Duplicate read marking, local realignment around indels, base quality score recalibration and variant calling were performed using GATK algorithms (Sentieon) for nuclear DNA. Variant data was annotated using a collection of tools (VcfAnno and VEP) with a variety of public and private variant databases including but not limited to gnomAD, ClinVar and HGMD. The median sequencing depth and coverage across the target regions for the tested sample were calculated based on MQ0 aligned reads. The sequencing run included in-process reference sample(s) for quality control, which passed our thresholds for sensitivity and specificity. The patient's sample was subjected to thorough quality control measures including assessments for contamination and sample mix-up.

Analysis in this study was limited to single-nucleotide variants, and small insertions/deletions and their combinations (INDELs) up to 220 bps within protein coding exons and exon-intron boundaries (± 20 bps). Copy number variations were excluded from the analysis. Performance metrics were as follows: WES: Median coverage 174x, >20x depth at target region 99.4%, >20x depth at *NRAP* gene 100%, sensitivity for SNVs 99.65%, indels 1–50 bps 99.1%, and specificity >99.9% and HQSA: median coverage 143x, >20x depth at target region 99.86%, >20x depth at *NRAP* gene 100%, sensitivity for SNVs 99.89%, indels 1–50 bps 99.2% and specificity >99.9%). Both assays have been validated in a CAP and ISO accredited laboratory (Blueprint Genetics, Finland).

#### *NRAP* variants

Since our aim to evaluate the role of potentially disease causing *NRAP* variants, the analysis was limited only to the variants with the highest potential to cause disease, specifically PTVs (nonsense, frameshift, canonical splice site, start lost) and missense variants as most of the synonymous and intronic variants are less likely to be disease causing. In addition, variants were included into further analysis only if no homozygous carriers were present in the Genome Aggregation Database control cohort (gnomAD) [17] and missense variants with 100 or less heterozygous individuals in gnomAD. Frequency of such high-quality variants were compared between patients with clinical or suspected dilated cardiomyopathy (DCM group), other cardiology indication (Non-DCM cardiac group consisting patients tested due diagnosis or suspicion inherited aortopathy, channelopathy or cardiomyopathy other than DCM) or any other clinical indication for panel or exome testing (Control group).

### Statistics

Comparisons between groups were performed with either Fisher’s exact or Chi-Square test for categorical variables and unpaired T-test for normally distributed continuous variables. Odds ratios (ORs) for DCM and non-DCM cardiac group *vs*. control group were calculated, and 95% confidence intervals (CIs) were determined using the conditional maximum likelihood/Fishers’ method. Normally distributed parameters are presented as mean ± standard deviation.

## Results

### Whole exome sequencing (WES) data set and *NRAP* variants

All variant calls from the *NRAP* gene were queried from the internal variant database in 31,639 individuals who underwent genetic testing using NGS-panels or direct WES approach. Of these patients, 577 were tested due to DCM or suspected DCM (DCM group), 5,150 due to suspicion of other monogenic cardiac disease (Non-DCM cardiac group) and 25,912 served as controls (control group).

### Enrichment of *NRAP* variants in DCM

We identified cases with two rare *NRAP* variants, of which at least one was a PTV in 11 out 577 (1.91%) patients in the DCM group but none were in either the non-DCM cardiac group or control group (Table 2). Frequency of such variant combination was significantly greater in the DCM group vs. controls (OR 1052, 95%CI 62–17876, p<0.0001; Table 3). Three of the patients had familial cardiomyopathy and eight had a sporadic disease. In these 11 individuals, four had a homozygous PTV, one had two heterozygous PTVs (phase unknown) and two were compound heterozygous for a PTV and a missense variant (*in trans*). In five patients, the phase of the *NRAP* variants was unknown. Two presumably unrelated patients had the same frameshift/missense variant combination (c.4371del, p.Thr1458Glnfs*36 and c.72G>C, p.Gln24His) and two had the same nonsense/missense variant combination (c.4504C>T, p.Arg1502* and c.72G>C, p.Gln24His). Thus, the p.(Gln24His) missense variant was observed altogether in four presumably unrelated patients. This variant may in fact lead to splicing defect as it affects the last nucleotide of the exon 1. Alamut Visual Splicing software v2.11 (Interactive Biosoftware, France) predicts that this variant either leads to loss of the native splice donor (NNSPLICE) or significant weakening of this site (SSF, MaxEnt). One patient had a start lost variant, *NRAP* p.(Met1?), which is expected to cause loss-of-function as there is an alternative out-of-frame start codon 5-bp down-stream from the wild type initiation codon.

**Table 2:** *NRAP* variants observed in the patients with dilated cardiomyopathy.

Patient	Variants	HGVS nomenclature	Variant type	Exon	gnomAD AC	SIFT	Cons.	ACMG Class
**1**	**10:115374685**	c.3099G>A, p.(Trp1033*)	**Nonsense**	**28/42**	**18**			**LP**
**2**	**10:115356904**	c.4371del, p.(Thr1458Glnfs*36)	**Frameshift**	**37/42**	**100**			**P**
	**10:115423570**	**c.72G>C, p.(Gln24His)**	**Missense**	**1/42**	**34**	**Delet. (0.01)**	**Full**	**P**
3	10:115356904	c.4371del, p.(Thr1458Glnfs*36)	Frameshift	37/42	100			P
	10:115423570	c.72G>C, p.(Gln24His)	Missense	1/42	34	Delet. (0.01)	Full	P
4	10:115400070	c.1344T>A, p.(Tyr448*)	Nonsense	14/42	35	Delet. (0.01)	Full	P
	10:115423593	c.49G>A, p.(Glu17Lys)	Missense	1/42	5			VUS
5	10:115356904	c.4371del, p.(Thr1458Glnfs*36)	Frameshift	37/42	100			P
	10:115355414	c.4504C>T, p.(Arg1502*)	Nonsense	38/42	95			P
**6**	**10:115423570**	**c.72G>C, p.(Gln24His)**	**Missense**	**1/42**	**34**	**Delet. (0.01)**	**Full**	**P**
	**10:115355414**	c.4504C>T, p.(Arg1502*)	**Nonsense**	**38/42**	**95**			**P**
7	10:115423570	c.72G>C, p.(Gln24His)	Missense	1/42	34	Delet. (0.01)	Full	P
	10:115355414	c.4504C>T, p.(Arg1502*)	Nonsense	38/42	95			P
**8**	**10:115356904**	c.4371del, p.(Thr1458Glnfs*36)	**Frameshift**	**37/42**	**100**			**P**
**9**	**10:115374675**	c.3109C>T, p.(Arg1037*)	**Nonsense**	**28/42**	**2**			**LP**
10	10:115364570	c.4025G>A, p.(Ser1342Asn)	Missense	35/42	3	Delet. (0.01)	Full	VUS
	10:115423640	c.2T>C, p.(Met1?)	Start lost	1/42	21			LP
**11**	**10:115400070**	c.1344T>A, p.(Tyr448*)	**Nonsense**	**14/42**	**35**			**P**

Genomic coordinates refer to human reference genome (GRCh37/hg19) and mutation nomenclature is based on GenBank accession NM_001261463.1 (NRAP). Homozygotes and compound heterozygous patients are marked with bold font. Cons, Conservation in mammals; Delet., Deleterious; GnomAD AC, Allele count in gnomAD reference population cohort; LP, Likely pathogenic; P, Pathogenic; VUS, Variant of Uncertain Significance. No homozygotes for these variants are present in the gnomAD reference population cohort.

**Table 3:** Enrichment of rare *NRAP* variants in patients with dilated cardiomyopathy (DCM).

	Control group	Non-DCM cardiac group	OR (95% CI), P-value	DCM group	OR (95% CI), p-value
Individuals (n)	25912	5150		577	
**Dominant hypothesis**					
Only one PTV variant	75 (0.29%)	24 (0.47%)	1.61 (1.02–2.56), p = 0.04	11 (1.91%)	6.71 (3.5–12.7), p<0.0001
Only one missense variant	698 (2.45%)	132 (2.56%)	1.05 (0.86–1.26), p = 0.65	10 (1.74%)	0.70 (0.4–1.3), p = 0.27
**Recessive hypothesis**					
Two missense variants	27 (0.10%)	1 (0.02%)	0.18 (0.02–1.35), p = 0.10	0 (0.0%)	0.81 (0.05–13.4), p = 0.89
One PTV + one missense	0	0	NA	6 (1.04%)	590 (33–10494), p<0.0001
Two PTV variants	0	0	NA	5 (0.87%)	407 (22–7575), p<0.0001
PTV + missense or 2 PTVs	0	0	NA	11 (1.91%)	1052(62–17876), p<0.0001

Control group patients underwent genetic testing due to non-cardiac reasons and non-DCM group patients due to cardiological reasons excluding patients with DCM or suspected DCM. DCM group includes patients tested with a DCM Panel or other panels because of confirmed or suspected DCM. Abbreviations: pts, patients; OR, odds ratio; 95% CI, 95% confidence interval, DCM, dilated cardiomyopathy, PTV, Protein-truncating variant (means here nonsense, frameshift variant, consensus splice site, start lost). Only variants with 100 or fewer heterozygous individuals in a gnomAD reference population cohort were included in calculations.

None of these 11 patients had an alternative molecular diagnosis identified in either large NGS panel (n = 9) or exome sequencing (n = 2). Six (55%) of the patients have had major endpoints, defined as history of cardiac transplantation (n = 2), death on waiting list for heart transplantation (n = 1) or during left ventricular assist device (LVAD) treatment (n = 2), and previous cardiac arrest (n = 1). The mean age at the time of the major endpoint was 22.8±19.4 years (Table 4). Four of these six patients had homozygous PTV in *NRAP* and one patient had two PTVs (phase unknown). Patients with two PTVs (n = 5) were younger at disease onset than patients with PTV + missense variant (n = 6) combination (19.6±20.4 vs. 48.3±12.3 years, p = 0.018). None of the patients had known skeletal muscle involvement.

**Table 4:** Clinical characteristics of patients with biallelic or potentially biallelic *NRAP* variants.

Patient	Age	Gender	Phenotype	LVEDD(mm)/EF%	Age at onset	Htx	Died	Other
1	19	F	DCM	NA	<19	Yes, at age of 19		
2	36	F	DCM	71/10-15%	NA		36	
3	31	M	DCM	70/13%	28			Mild LGE
4	57	M	DCM	NA	56			Maximum treatment for heart failure
5	4	F	DCM	58/20%	4	LVAD		
6	59	F	DCM	63/34%	46			
7	59	M	DCM	NA/30%	NA			LBBB
8	43	M	DCM	NA	22	Yes, at age 43		
9	53	F	DCM	NA	NA			History of cardiac arrest
10	48	M	DCM/HF	NA	NA			
11	2	M	DCM	NA	NA		2	

Age means age at last follow-up. Abbreviations: DCM, Dilated cardiomyopathy; EF, Ejection fraction; F, Female; HF, Heart failure; Htx, Heart transplantation; LBBB, Left bundle branch block; LGE, Late gadolinium enhancement at cardiac MRI; LVEDD, Left ventricular end-diastolic diameter in mm; M, Male; NA, Not available or not known.

A single heterozygous PTV without another rare *NRAP* variant was observed in 11 patients (1.91%) and they were also enriched in the DCM group (OR 6.71, 95% CI 3.5–12.7, p<0.0001; Table 3). The single heterozygous PTV group excludes all patients with two rare *NRAP* variants as defined earlier. However, one of these patients also had another moderately rare (500 heterozygotes in gnomAD) missense variant in *NRAP* (c.2963A>C, p.(Gln988Pro); phase unknown) in addition to a start-lost variant. The patient had no alternative molecular diagnosis in established cardiomyopathy genes. Of the 11 patients with only one heterozygous PTV in *NRAP*, three had another molecular diagnosis including three PTVs affecting A-band of *TTN* and one had an additional frameshift variant in *DSP*.

### Familial segregation

We were able to recruit two out of the three probands with familial disease and one with sporadic disease for additional screening. Co-segregation was assessed from 18 family members who underwent screening of familial variants and clinical history, as well as and clinical evaluation including ECG and echocardiography. Cardiac MRI was performed as needed.

**Fig 1:**
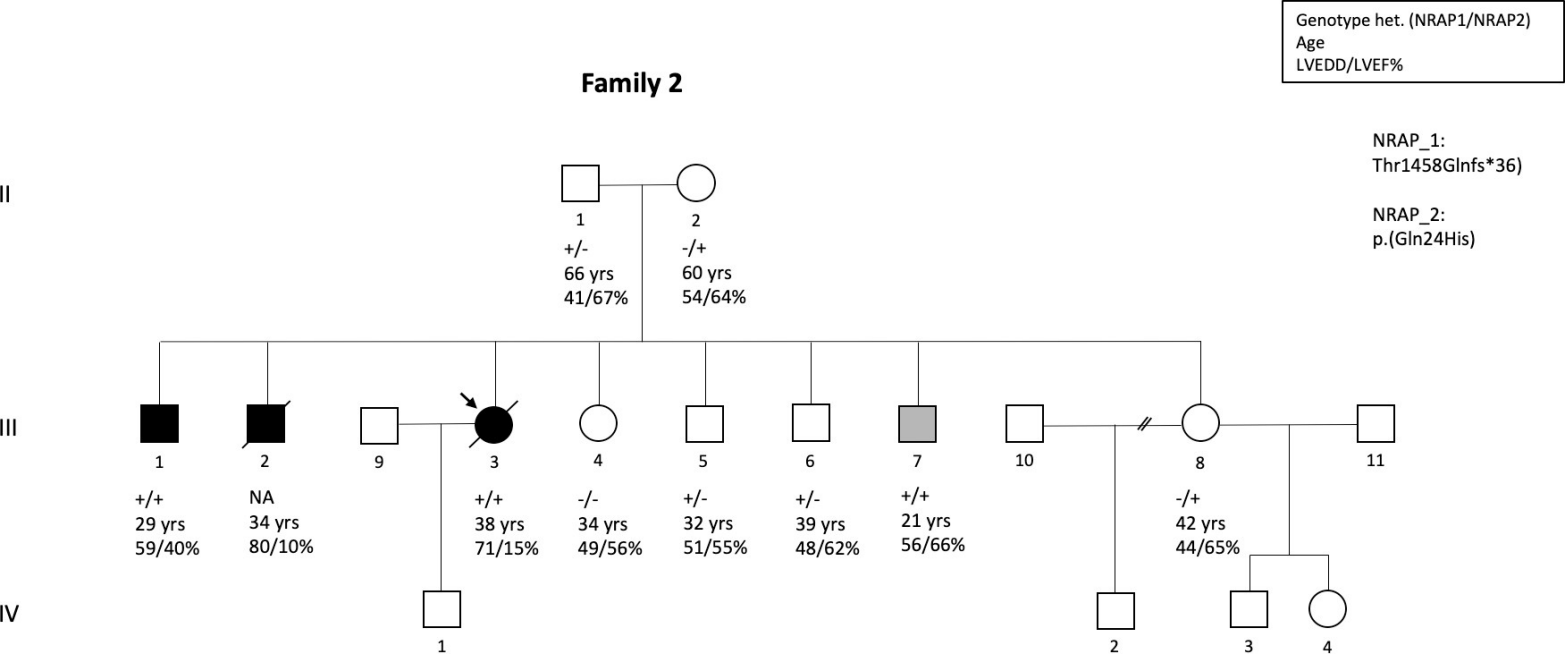
Pedigree of the family-2 where the index patient and her affected brother were compound heterozygous for c.4371del, p.(Thr1458Glnfs*36) and c.72G>C, p.(Gln24His) in *NRAP* similarly as her 21-year brother who were on medication initiated before the results of genetic testing were available due to borderline imaging findings suggesting cardiomyopathy. He did not fulfill diagnostic criteria of DCM at the time of the study. DNA was not available from one affected individual who died for DCM at age of 34. All family members who were heterozygous only for the other variant or were homozygous for the wild type allele were unaffected.

In family 2, the proband died at the age of 38 years from severe biventricular heart failure. She was compound heterozygous for c.4371del, p.(Thr1458Glnfs*36) and c.72G>C, p.(Gln24His) in *NRAP* (Table 2, Fig 1). At the time of last imaging study, her LVEDD was 71 mm, LVEF was 13% and RVEF was 17%, and she had elevated levels of TnI and proBNP and a widened QRS (132 ms). One of the proband’s brothers was diagnosed with DCM at the age of 24 years and he died at age of 34 years of severe biventricular heart failure. No DNA sample was available from this individual for genetic testing. Two of the family members were compound heterozygous for the same variants. One had a diagnosis of mild DCM at age 20 and no progression since initiating ACE inhibitor treatment, and the other had upper normal LV size despite of treatment initiation at the age of 21 years. All five heterozygous siblings and one with wild type allele were healthy. The parents of the proband were both heterozygous for one the variants and had normal echocardiography.

**Fig 2:**
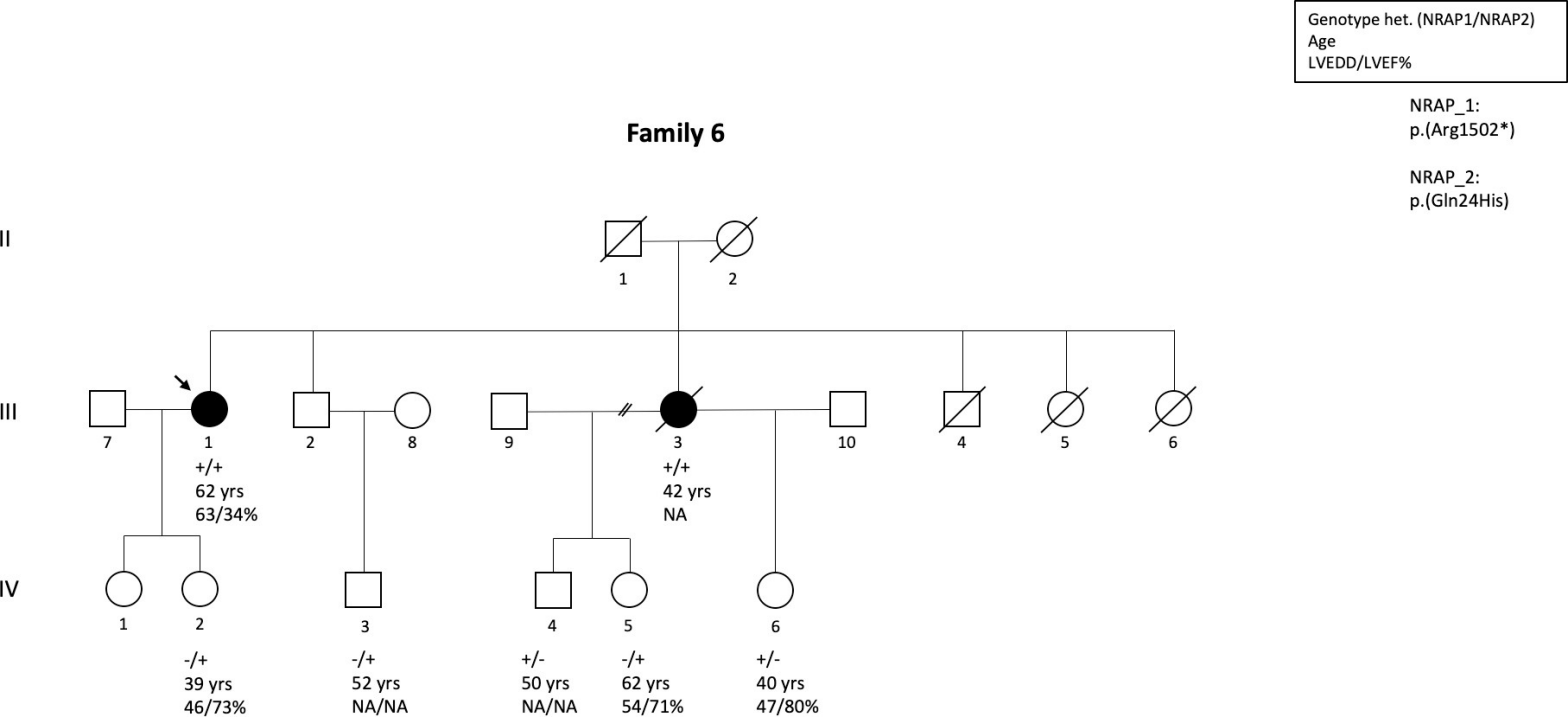
Pedigree of the family-6 where the index patient and her affected brother were compound heterozygous for c.4504C>T, p.(Arg1502*) and c.72G>C, p.(Gln24His) in *NRAP*. All family members who were heterozygous only for the other variant were unaffected.

In family 6, the proband was diagnosed with DCM at the age of 47 years due to dilated LV and reduced LV function (LVEDD 63mm, EF 34%). She was compound heterozygous for c.4504C>T, p.(Arg1502*) and c.72G>C, p.(Gln24His) in *NRAP* (Table 2, Fig 2). Mild improvement in LV size and function were observed with medical treatment. The proband’s sister died at the age 40 years due to DCM. She was an obligate compound heterozygote for the same variants as the proband, which was discovered after the testing of her children. Three of the proband’s siblings have died during childhood, but no samples were available from any of them for genetic testing. In the extended family, two heterozygous individuals and two with wild type alleles were healthy. The proband’s parents, who were both obligatory heterozygotes for one variant, had no known cardiomyopathy and had a normal life span.

**Fig 3:**
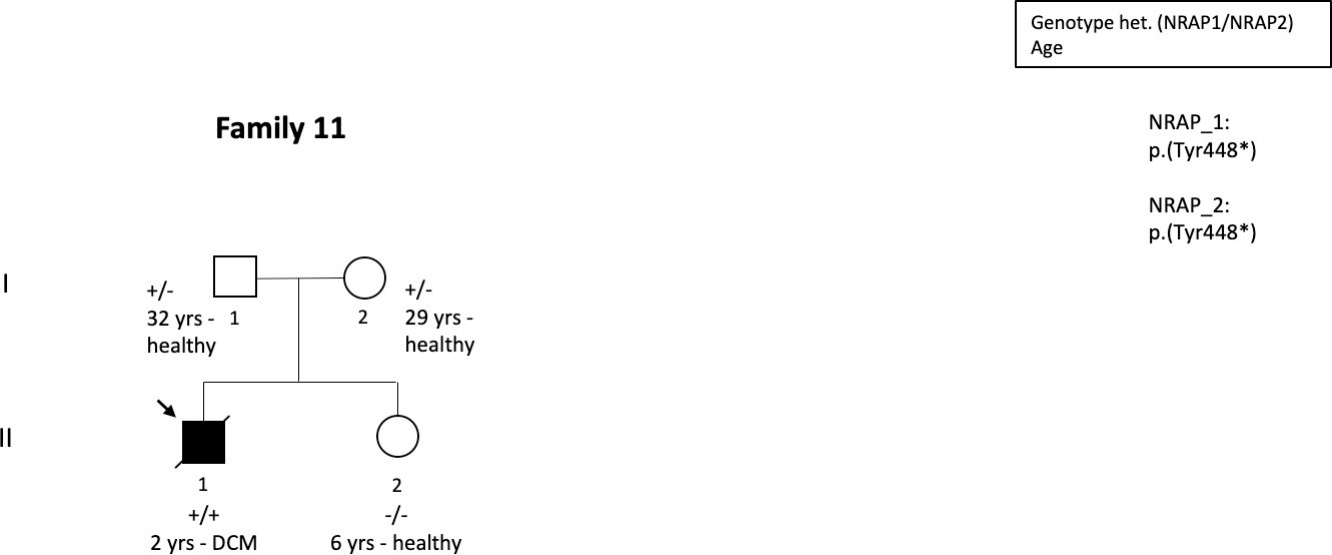
Pedigree of the family-11 where the index patient was homozygous for c.1344T>A, p.(Tyr448*) in *NRAP* whereas all other family members who were heterozygous for the variant or had homozygous wild type allele were unaffected.

In family 11, the proband was diagnosed with DCM at age 2 due to dilated LV and reduced LV function. The patient was homozygous for c.1344T>A, p.(Tyr448*) in *NRAP* (Table 2, Fig 3). He received LVAD soon after hospitalization due to severe heart failure but he died before planned transplantation. The proband’s parents were heterozygotes for the variant and his older sister was homozygous for a wild type allele. All family members were healthy.

### Estimating frequency of biallelic protein truncating *NRAP* variants in general population

As we discovered significant new evidence supporting the role of biallelic *NRAP* variants in DCM, we decided to further estimate the potential contribution of this gene on DCM at a global scale. We queried the count of *NRAP*-PTV in gnomAD reference population v2.1.1. In total 733 high-quality PTVs were present in the database. The average number of alleles reported at these positions was 233,756 indicating a cumulative allele frequency of 0.31%. Thus, the probability of homozygosity or compound heterozygosity is approximately 0.000983% at the individual level. This is equal to 1 case per 101,700 individuals if we assume that only PTV variants would be disease causing.

## Discussion

Our data suggest that the variants in the *NRAP* gene are associated with DCM and may explain up to 1.91% of DCM cases in an unselected clinical cohort consisting of patients with either clinically diagnosed DCM or suspected DCM. Because small disease cohorts may provide inaccurate estimates due to non-random inclusion and pure coincidence, we decided to estimate the prevalence of potentially biallelic *NRAP*-PTV in a large population data set (gnomAD). This analysis yielded a frequency of 0.000983%, equal to 1 case per 101,700 individuals. If all of these variants were fully penetrant, *NRAP* might explain up to 0.34%-2.03% of all DCM cases when relying on variable (1:3,000 to 1:500) estimates of DCM prevalence in the general population. Thus, our DCM patient cohort and population data cohort provide essentially similar estimates of the contribution of *NRAP* in DCM.

In general, non-syndromic familial cardiomyopathies follow dominant inheritance [1,18]. In 2016, the *ALPK3* gene was discovered to be a rare cause of a recessive pediatric cardiomyopathy, which typically presents with DCM and non-compaction that progress to hypertrophic cardiomyopathy and possibly some syndromic features [19]. Some other genes such as *GATAD1* [20], *PLEKHM2* [21], and *PPCS* [22] have been shown to associate with recessive non-syndromic DCM. However, not much evidence has been gathered after initial reports, possibly reflecting the rarity of such gene-to-phenotype associations. Classic genes encoding cardiac desmosome proteins initially connected to ARVC/arrhythmogenic cardiomyopathy are now considered established causes of the DCM phenotype [10,23]. Notably, some variants in *DSG2* gene cause recessive ARVC that may be difficult to distinguish from DCM [24]. However, based on the numbers of reported patients and mutation database submissions (e.g. ClinVar) of patients carrying variants in previously described recessive cardiomyopathy genes, it seems likely that *NRAP* has a more prominent contribution to the etiology.

Previous reports involving *NRAP* gene did not include segregation analysis [13] had insufficient data obtained from the family studies to fully support segregation or were inconsistent with co-segregation [12,14,15]. In the first study suggesting association of *NRAP* with cardiomyopathy, the proband’s 35-year-old brother who was homozygous for PTV in *NRAP* was considered unaffected while being asymptomatic and having normal echocardiography and ECG [12]. Thus, the authors concluded that *NRAP* may be a low penetrance genetic risk factor for DCM even though the previous observation can also be explained by age-dependent penetrance of cardiomyopathies. Later, Ahmed et al. published a consanguineous pedigree in which the index patient was a baby girl who presented at the age of 13 months with heart failure, easy fatigability, weakness, irritability, and shortness of breath and was diagnosed with DCM [15]. Whole exome sequencing revealed that her healthy 33-year-old father was homozygous for the same frameshift variant identified in the proband whereas the mother was heterozygous. The proband’s family history included one stillbirth and another brother who was diagnosed with cardiomyopathy at the age of 12 months and died at 17 months without a molecular diagnosis (samples were not available for genetic testing). Otherwise the extended pedigree did not reveal any known cardiomyopathy cases, which also suggests recessive inheritance. Our previous study reported a family in which the index patient who was diagnosed with DCM at the age of 3 years [14]. The proband was homozygous for *NRAP* p.(Tyr448*). Three family members were heterozygous for the variant and one had a homozygous wildtype allele, and all of them were considered healthy. Individuals who are heterozygous for a single LoF variant in *NRAP* are cardiologically healthy in all previously published reports as well as in our study suggesting that *NRAP* does not cause dominantly inherited monogenic DCM. However, we cannot exclude the possibility that it would increase susceptibility to cardiomyopathy even when heterozygous due to observed enrichment of single LoF variants in our DCM cohort.

*NRAP* seems to associate with severe DCM as the proportion of patients with major cardiac endpoint (death, cardiac arrest, transplant and LVAD) is similar or higher compared to *LMNA* related cardiac laminopathy (54.5% vs. 58.3%) [25]. *NRAP* patients also seem to have an earlier onset of major cardiac end-points when compared to cardiac laminopathy or DCM in general (22.8±19.4 vs. 51.0±8.7 and was 59.0±14.2 years) [25]. In addition, the rate of cardiac transplantation and LVAD utilization was higher in our *NRAP* group compared to a Norwegian *LMNA* cohort (45% vs. 33%) [26].

Our data also suggest that two PTVs in *NRAP* cause more severe disease than PTV + missense combination in *NRAP*. There are no previous observations on the PTV + missense variant combination in DCM, thus further studies are needed to confirm whether the previous assumption is correct. Given that our data did not show enrichment of potentially biallelic missense variants in the DCM group, these variants may not contribute to the DCM pathogenesis alone. However, at this time we cannot exclude the possibility that a small proportion of biallelic missense variants are disease causing alone.

*NRAP* appear to play important role in myocardial architecture and sarcomere function, supporting the biological plausibility of our findings. The *NRAP* gene on chromosome 10q25.3 encodes the nebulin related anchoring protein. This protein is involved in anchoring terminal actin filaments to the membrane, tension transmission from myofibrils to extracellular matrix, as well as having a significant role in myofibrillogenesis during cardiomyocyte development, and it is involved in the sarcomeric contraction cycle in adult heart [27,28]. The N-terminal LIM domain of NRAP interacts with α-actinin and talin [29,30], while the domain with single repeats interacts also with actin, the Kelch-like family member 41 (*KLHL41*) [31], and cysteine and glycine-rich protein 3 (*CSRP3*) [27], and the C-terminal super repeats interact with filamin C (*FLNC*) [31] and vinculin (*VCL*) [29]. Experimentally, upregulation of NRAP expression was observed in DCM mice models and human DCM patients [27,32]. This has been suggested to be an adaptive response to correct for disorganized actin thin filament architecture at intercalated disc junctions. NRAP is expressed in the myocardium and in striated muscle. Truszkowska et al. previously reported an absence of NRAP protein in the myocardium of a DCM proband with biallelic PTV in *NRAP* whileNRAP protein was clearly present in a control heart [12].

### Study limitations

In one of the three probands with familial DCM, we were unable to obtain samples from the parents and other family members to further prove segregation of the phenotype with the genotype. Similarly, DNA samples were available only in one of the eight probands with sporadic DCM. Even though the data supported recessive inheritance since heterozygous individuals were unaffected, more thorough segregation studies would have brought depth to the scientific message especially by clarifying penetrance of *NRAP* related DCM. Moreover, no functional studies were carried out, nor animal models were generated for any of the identified variants. Four of the patients carried the same splice region missense variant, *NRAP* p.(Gln24His), but we did not perform transcriptional analysis to determine this variant's effect on splicing that would have increase our understanding on disease mechanisms. None of the *NRAP* variants detected via NGS were confirmed with Sanger sequencing since all of them had high variant call quality score, fulfilled several other quality control criteria for true positive call, and the reporting followed CLIA/CAP/ISO-15189 approved policy. This study provides the first statistical association between the *NRAP* gene and DCM without mechanistic insights or evidence that have been partially provided in the initial case reports.

The results of this study demonstrate significant enrichment of *NRAP* variants in DCM patients with severe clinical events and their co-segregation in multiple families support an inclusion of *NRAP* in genetic testing of cardiomyopathies.

## Supporting information

S1 File (XLSX)

## References

[1] Elliott P, Andersson B, Arbustini E, Bilinska Z, Cecchi F, Charron P, et al Classification of the cardiomyopathies: A position statement from the european society of cardiology working group on myocardial and pericardial diseases. Eur Heart J. 2008;29: 270–276. 10.1093/eurheartj/ehm342 17916581

[2] Codd MB, Sugrue DD, Gersh BJ, Melton LJ. Epidemiology of idiopathic dilated and hypertrophic cardiomyopathy. A population-based study in Olmsted County, Minnesota, 1975–1984. Circulation. 1989;80: 564–72. 10.1161/01.cir.80.3.564 2766509

[3] Mestroni L, Maisch B, McKenna WJ, Schwartz K, Charron P, Rocco C, et al Guidelines for the study of familial dilated cardiomyopathies. Eur Heart J. 1999;20: 93–102. 10.1053/euhj.1998.1145 10099905

[4] McNally EM, Golbus JR, Puckelwartz MJ. Genetic mutations and mechanisms in dilated cardiomyopathy. J Clin Invest. 2013;123: 19–26. 10.1172/JCI62862 23281406 PMC3533274

[5] Cahill TJ, Ashrafian H, Watkins H. Genetic Cardiomyopathies Causing Heart Failure. Circ Res. 2013;113: 660–675. 10.1161/CIRCRESAHA.113.300282 23989711

[6] Hershberger RE, Hedges DJ, Morales A. Dilated cardiomyopathy: the complexity of a diverse genetic architecture. Nat Rev Cardiol. 2013;10: 531–547. 10.1038/nrcardio.2013.105 23900355

[7] Mestroni L, Taylor MRG. Genetics and genetic testing of dilated cardiomyopathy: a new perspective. Discov Med. 2013;15: 43–9. 23375013 PMC3929942

[8] Pugh TJ, Kelly MA, Gowrisankar S, Hynes E, Seidman MA, Baxter SM, et al The landscape of genetic variation in dilated cardiomyopathy as surveyed by clinical DNA sequencing. Genet Med. 2014;16: 601–608. 10.1038/gim.2013.204 24503780

[9] Herman DS, Lam L, Taylor MRG, Wang L, Teekakirikul P, Christodoulou D, et al Truncations of titin causing dilated cardiomyopathy. N Engl J Med. 2012;366: 619–28. 10.1056/NEJMoa1110186 22335739 PMC3660031

[10] Akinrinade O, Ollila L, Vattulainen S, Tallila J, Gentile M, Salmenperä P, et al Genetics and genotype-phenotype correlations in Finnish patients with dilated cardiomyopathy. Eur Heart J. 2015;36: 2327–37. 10.1093/eurheartj/ehv253 26084686 PMC4561350

[11] Walsh R, Thomson KL, Ware JS, Funke BH, Woodley J, McGuire KJ, et al Reassessment of Mendelian gene pathogenicity using 7,855 cardiomyopathy cases and 60,706 reference samples. Genet Med. 2017;19: 192–203. 10.1038/gim.2016.90 27532257 PMC5116235

[12] Truszkowska GT, Bilińska ZT, Muchowicz A, Pollak A, Biernacka A, Kozar-Kamińska K, et al Homozygous truncating mutation in NRAP gene identified by whole exome sequencing in a patient with dilated cardiomyopathy. Sci Rep. 2017;7: 1–5. 10.1038/s41598-016-0028-x 28611399 PMC5469774

[13] Monies D, Abouelhoda M, AlSayed M, Alhassnan Z, Alotaibi M, Kayyali H, et al The landscape of genetic diseases in Saudi Arabia based on the first 1000 diagnostic panels and exomes. Hum Genet. 2017;136: 921–939. 10.1007/s00439-017-1821-8 28600779 PMC5502059

[14] Vasilescu C, Ojala TH, Brilhante V, Ojanen S, Hinterding HM, Palin E, et al Genetic Basis of Severe Childhood-Onset Cardiomyopathies. J Am Coll Cardiol. 2018;72: 2324–2338. 10.1016/j.jacc.2018.08.2171 30384889

[15] Ahmed HA, Al-ghamdi S, Mutairi F Al. Dilated cardiomyopathy in a child with truncating mutation in NRAP gene. JBCGenetics. 2018;1: 77 LP– 80. Available: https://www.jbcgenetics.com//?mno = 17290.

[16] Martin AR, Williams E, Foulger RE, Leigh S, Daugherty LC, Niblock O, et al PanelApp crowdsources expert knowledge to establish consensus diagnostic gene panels. Nat Genet. 2019;51: 1560–1565. 10.1038/s41588-019-0528-2 31676867

[17] Karczewski KJ, Francioli LC, Tiao G, Cummings BB, Alföldi J, Wang Q, et al Variation across 141,456 human exomes and genomes reveals the spectrum of loss-of-function intolerance across human protein-coding genes. bioRxiv. 2019 10.1101/531210

[18] Haas J, Frese KS, Peil B, Kloos W, Keller A, Nietsch R, et al Atlas of the clinical genetics of human dilated cardiomyopathy. Eur Heart J. 2015;36: 1123–1135. 10.1093/eurheartj/ehu301 25163546

[19] Almomani R, Verhagen JMA, Herkert JC, Brosens E, van Spaendonck-Zwarts KY, Asimaki A, et al Biallelic Truncating Mutations in ALPK3 Cause Severe Pediatric Cardiomyopathy. J Am Coll Cardiol. 2016;67: 515–525. 10.1016/j.jacc.2015.10.093 26846950

[20] Theis JL, Sharpe KM, Matsumoto ME, Chai HS, Nair AA, Theis JD, et al Homozygosity mapping and exome sequencing reveal GATAD1 mutation in autosomal recessive dilated cardiomyopathy. Circ Cardiovasc Genet. 2011;4: 585–9. 10.1161/CIRCGENETICS.111.961052 21965549 PMC3248690

[21] Muhammad E, Levitas A, Singh SR, Braiman A, Ofir R, Etzion S, et al PLEKHM2 mutation leads to abnormal localization of lysosomes, impaired autophagy flux and associates with recessive dilated cardiomyopathy and left ventricular noncompaction. Hum Mol Genet. 2015;24: 7227–40. 10.1093/hmg/ddv423 26464484 PMC4664165

[22] Iuso A, Wiersma M, Schüller HJ, Pode-Shakked B, Marek-Yagel D, Grigat M, et al Mutations in PPCS, Encoding Phosphopantothenoylcysteine Synthetase, Cause Autosomal-Recessive Dilated Cardiomyopathy. Am J Hum Genet. 2018;102: 1018–1030. 10.1016/j.ajhg.2018.03.022 29754768 PMC5992122

[23] Garcia-Pavia P, Syrris P, Salas C, Evans A, Mirelis JG, Cobo-Marcos M, et al Desmosomal protein gene mutations in patients with idiopathic dilated cardiomyopathy undergoing cardiac transplantation: a clinicopathological study. Heart. 2011;97: 1744–1752. 10.1136/hrt.2011.227967 21859740

[24] Qadri S, Anttonen O, Viikilä J, Seppälä EH, Myllykangas S, Alastalo T-P, et al Case reports of two pedigrees with recessive arrhythmogenic right ventricular cardiomyopathy associated with homozygous Thr335Ala variant in DSG2. BMC Med Genet. 2017;18: 86 10.1186/s12881-017-0442-3 28818065 PMC5561604

[25] Ollila L, Nikus K, Holmström M, Jalanko M, Jurkko R, Kaartinen M, et al Clinical disease presentation and ECG characteristics of LMNA mutation carriers. Open Hear. 2017;4: e000474 10.1136/openhrt-2016-000474 28123761 PMC5255551

[26] Hasselberg NE, Haland TF, Saberniak J, Brekke PH, Berge KE, Leren TP, et al Lamin A/C cardiomyopathy: young onset, high penetrance, and frequent need for heart transplantation. Eur Heart J. 2018;39: 853–860. 10.1093/eurheartj/ehx596 29095976 PMC5939624

[27] Ehler E, Horowits R, Zuppinger C, Price RL, Perriard E, Leu M, et al Alterations at the intercalated disk associated with the absence of muscle LIM protein. J Cell Biol. 2001;153: 763–72. 10.1083/jcb.153.4.763 11352937 PMC2192386

[28] Henderson CA, Gomez CG, Novak SM, Mi-Mi L, Gregorio CC. Overview of the muscle cytoskeleton. Compr Physiol. 2017;7: 891–944. 10.1002/cphy.c160033 28640448 PMC5890934

[29] Luo G, Herrera AH, Horowits R. Molecular Interactions of N-RAP, a Nebulin-Related Protein of Striated Muscle Myotendon Junctions and Intercalated Disks. Biochemistry. 1999;38: 6135–6143. 10.1021/bi982395t 10320340

[30] Zhang JQ, Elzey B, Williams G, Lu S, Law DJ, Horowits R. Ultrastructural and biochemical localization of N-RAP at the interface between myofibrils and intercalated disks in the mouse heart. Biochemistry. 2001;40: 14898–906. 10.1021/bi0107445 11732910

[31] Lu S, Carroll SL, Herrera AH, Ozanne B, Horowits R. New N-RAP-binding partners α-actinin, filamin and Krp1 detected by yeast two-hybrid screening: Implications for myofibril assembly. J Cell Sci. 2003;116: 2169–78. 10.1242/jcs.00425 12692149

[32] Perriard JC, Hirschy A, Ehler E. Dilated cardiomyopathy: A disease of the intercalated disc? Trends Cardiovasc Med. 2003;13: 30–38. 10.1016/s1050-1738(02)00209-8 12554098

