# Supplementary material for: Biallelic loss-of-function in NRAP is a cause of recessive dilated cardiomyopathy
Source: PLoS One. 2021 Feb 3;16(2):e0245681. doi: 10.1371/journal.pone.0245681 (PMC7857588; doi:10.1371/journal.pone.0245681)
Supplement: Attachment — Submitted filename: response to reviewers PONE-D-20-18740.docx [file pone.0245681.s002.docx]

PONE-D-20-18740
Biallelic loss-of-function in NRAP is a common cause of recessive dilated cardiomyopathy
PLOS ONE

Dear Dr. Koskenvuo,

Thank you for submitting your manuscript to PLOS ONE. After careful consideration, we feel that it has merit but does not fully meet PLOS ONE’s publication criteria as it currently stands. Therefore, we invite you to submit a revised version of the manuscript that addresses the points raised during the review process.

**Ed_1**. Address reviewer 1's concern on the lack of detail on the clinical phenotyping of patients and include more details on these.

RESPONSE: << OK. Please see a new Table 4, which contain all available clinical information>>.

**Ed_2**. Provider more details for the pipeline used in the bioinformatics analysis.

RESPONSE: <<We think it is out-of-the-scope of the current work but basic information on both whole exome (<https://blueprintgenetics.com/tests/whole-exome-sequencing/whole-exome-plus/>) and clinical exome assays (<https://blueprintgenetics.com/tests/panels/cardiology/comprehensive-cardiology-panel/>) are provided at laboratory’s website when selecting: 1) Test Strength and Limitations, 2) Test Performance, 3) Bioinformatics and Clinical Interpretation.>>.

**Ed_3**. Address reviewer's comments on improvements for Tables 1 and 2.

RESPONSE: << Done>>.

**Ed_4**. Fix discrepancies in numbers identified by reviewer 2.

RESPONSE: << Done. Please see response to Rev2.1 and Rev2.2>>.

**Ed_5.** Respond to reviewer 4's comments related to exome sequencing and lack of detection of other DCM genes.

RESPONSE: << Done, Please see our response Rev 4.1>>.

**Ed_6.** Consider adding in the reference suggested by Reviewer 4.

RESPONSE: <<It was already cited in the original manuscript draft>>.

**Ed_7.** Provide greater detail on the inclusion and exclusion criteria for variants.  See comments from reviewers.  Also address how these variants meet ACMG criteria.

RESPONSE: << This is now provided in the methods/NRAP variants: ” Since we were aiming to evaluate the role of potentially disease causing *NRAP* variants, the analysis was limited only to the variants with the highest potential to cause disease, specifically PTVs (nonsense, frameshift, canonical splice site, start lost) and missense variants as most of the synonymous and intronic variants are less likely to be disease causing. In addition, variants were included into further analysis only if no homozygous carriers were present in the Genome Aggregation Database control cohort (gnomAD) [17] and missense variants with 100 or less heterozygous individuals in gnomAD.”

The variants were classified according to ACMG criteria and classifications are now present in Table 2>>.

**Ed_8.** Address the other points raised by the reviewers.

RESPONSE: << Done>>.

Journal_Requirements_1. Please ensure that your manuscript meets PLOS ONE's style requirements, including those for file naming. The PLOS ONE style templates can be found at

<https://journals.plos.org/plosone/s/file?id=wjVg/PLOSOne_formatting_sample_main_body.pdf> and

<https://journals.plos.org/plosone/s/file?id=ba62/PLOSOne_formatting_sample_title_authors_affiliations.pdf>

RESPONSE: << Done >>.

Journal_Requirements_2. Thank you for stating the following in the Competing Interests section:

"Drs. Koskenvuo, Saarinen, Ahonen, Tommiska, Seppälä, Tuupanen, Kangas-Kontio, Schleit, Hathaway, Kytölä, Muona, Sistonen, Salmenperä, Gentile, Paananen, Myllykangas, Alastalo are full-time employees of Blueprint Genetics, which offers genetic diagnostics for cardiomyopathies. All other authors have reported that they have no relationships relevant to the contents of this paper to disclose."

We note that one or more of the authors are employed by a commercial company: Blueprint Genetics, a Quest Diagnostics Company.

Journal_Requirements_2.1. Please provide an amended Funding Statement declaring this commercial affiliation, as well as a statement regarding the Role of Funders in your study. If the funding organization did not play a role in the study design, data collection and analysis, decision to publish, or preparation of the manuscript and only provided financial support in the form of authors' salaries and/or research materials, please review your statements relating to the author contributions, and ensure you have specifically and accurately indicated the role(s) that these authors had in your study. You can update author roles in the Author Contributions section of the online submission form.

Please also include the following statement within your amended Funding Statement.

“The funder provided support in the form of salaries for authors [insert relevant initials], but did not have any additional role in the study design, data collection and analysis, decision to publish, or preparation of the manuscript. The specific roles of these authors are articulated in the ‘author contributions’ section.”

If your commercial affiliation did play a role in your study, please state and explain this role within your updated Funding Statement.

RESPONSE: << Done. The following text is now present in the manuscript: “

Drs. Koskenvuo, Saarinen, Ahonen, Tommiska, Seppälä, Tuupanen, Kangas-Kontio, Schleit, Hathaway, Kytölä, Muona, Sistonen, Salmenperä, Gentile, Paananen, Myllykangas, Alastalo are full-time employees of Blueprint Genetics, a Quest Diagnostics Company, which offers genetic diagnostics for cardiomyopathies. The funder provided support in the form of salaries for authors [JWK, IS, SA, JT, ST, TKK, JS, JH, VK, MM, JS, PS, MG, JP, SM, TPA], but did not have any additional role in the study design, data collection and analysis, decision to publish, or preparation of the manuscript. The specific roles of these authors are articulated in the ‘author contributions’ section. All other authors have reported that they have no relationships relevant to the contents of this paper to disclose."

>>.

Journal_Requirements_2.2. Please also provide an updated Competing Interests Statement declaring this commercial affiliation along with any other relevant declarations relating to employment, consultancy, patents, products in development, or marketed products, etc.

Within your Competing Interests Statement, please confirm that this commercial affiliation does not alter your adherence to all PLOS ONE policies on sharing data and materials by including the following statement: "This does not alter our adherence to  PLOSONE policies on sharing data and materials.” (as detailed online in our guide for authors [http://journals.plos.org/plosone/s/competing-interests](about:blank)) . If this adherence statement is not accurate and  there are restrictions on sharing of data and/or materials, please state these. Please note that we cannot proceed with consideration of your article until this information has been declared.

Please include both an updated Funding Statement and Competing Interests Statement in your cover letter. We will change the online submission form on your behalf.

Please know it is PLOS ONE policy for corresponding authors to declare, on behalf of all authors, all potential competing interests for the purposes of transparency. PLOS defines a competing interest as anything that interferes with, or could reasonably be perceived as interfering with, the full and objective presentation, peer review, editorial decision-making, or publication of research or non-research articles submitted to one of the journals. Competing interests can be financial or non-financial, professional, or personal. Competing interests can arise in relationship to an organization or another person. Please follow this link to our website for more details on competing interests: [http://journals.plos.org/plosone/s/competing-interests](about:blank)

RESPONSE: << The commercial connection does not alter our adherence to  PLOSONE policies on sharing data and materials. Please see the new supplementary file 1, which consists all called rare *NRAP* variants in the cohort. We have modified the funding statement and COIs as requested >>.

Journal_Requirements_3. In your Data Availability statement, you have not specified where the minimal data set underlying the results described in your manuscript can be found. PLOS defines a study's minimal data set as the underlying data used to reach the conclusions drawn in the manuscript and any additional data required to replicate the reported study findings in their entirety. All PLOS journals require that the minimal data set be made fully available. For more information about our data policy, please see <http://journals.plos.org/plosone/s/data-availability>.

Upon re-submitting your revised manuscript, please upload your study’s minimal underlying data set as either Supporting Information files or to a stable, public repository and include the relevant URLs, DOIs, or accession numbers within your revised cover letter. For a list of acceptable repositories, please see <http://journals.plos.org/plosone/s/data-availability#loc-recommended-repositories>. Any potentially identifying patient information must be fully anonymized.

Important: If there are ethical or legal restrictions to sharing your data publicly, please explain these restrictions in detail. Please see our guidelines for more information on what we consider unacceptable restrictions to publicly sharing data: <http://journals.plos.org/plosone/s/data-availability#loc-unacceptable-data-access-restrictions>. Note that it is not acceptable for the authors to be the sole named individuals responsible for ensuring data access.

We will update your Data Availability statement to reflect the information you provide in your cover letter.

RESPONSE: << Please see the new supplementary file 1, which consists all called rare *NRAP* variants in the cohort. Using this information, one can re-perform the statistics presented in the study >>.

Journal_Requirements_4. Please include captions for your Supporting Information files at the end of your manuscript, and update any in-text citations to match accordingly. Please see our Supporting Information guidelines for more information: [http://journals.plos.org/plosone/s/supporting-information](about:blank).

RESPONSE: << Please see the new supplementary file 1, which consists all called rare *NRAP* variants in the cohort.>>.

**Reviewer 1**

In this manuscript by Koskenvuo and colleagues utilize a large genetic testing database to identify a number of rare variants in NRAP associated with DCM. Overall, this is an appropriately concise report that is well written. It attempts to tackle a common, yet challenging problem – what is the cause of genotype negative DCM?

The size of the cohort analyzed, and the limited co-segregation present are strengths of this paper. However, there are a number of points which should be expanded or clarified in my opinion.

**Rev1_1**: In the introduction, a brief explanation of the physiologic role of NRAP would be helpful. Is it expressed in the heart? Solely in the heart? Would its expression pattern lend itself to a cardiac myocardium only disease?

RESPONSE: << We tried to keep the introduction concise and present physiologic background of this protein in the discussion section (see last chapter) since this study is more about genetic etiology than functional study evaluating NRAP protein function at cellular level. It is difficult to predict final consequence of mutated gene by expression pattern since expression is dependent on age (e.g. embryonically expressed genes vs adult, organ specific (myocardium is working all the time but skeletal muscle only when exercised. By expression pattern, it is possible that it would also associate to skeletal muscle involvement (<https://gtexportal.org/home/gene/NRAP>). However, none of the patients had known issued with skeletal muscle.>>

**Rev1_2:** A major limitation of this manuscript is the lack of clinical phenotyping of the patients with DCM. This should be addressed. What is the evidence that the cohort that was screened had non-ischemic, congenital DCM? This is even more of any issue for the NRAP-positive individuals.

RESPONSE: << We have added a new table (Table 4), which includes all available data on phenotypes. All individuals who were screened had a suspicion of familial dilated cardiomyopathy or DCM with genetic etiology. If it would be the case that some patients would not have even DCM at DCM group, it would only dilute the enrichment. Thus, this study set-up is not prone to find coincidental associations.>>

**Rev1_3:** The bioinformatic pipeline used to identify these variants is not well delineated. How were other variants that localized to genes which are known to cause DCM excluded as possibilities?

RESPONSE: << We included all patients who underwent genetic testing due to DCM or suspected DCM to the DCM group despite of molecular diagnosis status. Importantly, none of the 11 patients with two *NRAP* variants had an alternative molecular diagnosis identified in either large NGS panel (n=9) or exome sequencing (n=2). This is mentioned in the results section.

The pipeline and related methodologies including wet lab and clinical interpretation represent accredited NGS services. Please see information on both whole exome (<https://blueprintgenetics.com/tests/whole-exome-sequencing/whole-exome-plus/>) and clinical exome assays (<https://blueprintgenetics.com/tests/panels/cardiology/comprehensive-cardiology-panel/>) at laboratory’s website by selecting: 1) Test Strength and Limitations, 2) Test Performance, 3) Bioinformatics and Clinical Interpretation.>>

**Rev1_4:** Were copy number variants identified? This is important to address as there are CNVs associated with DCM (1p36 deletion syndrome) which may confound this data.

RESPONSE: << Roughly half of the patients were evaluated for CNVs and only few positive cases were found e.g. *DMD* deletions. This is out of this project’s scope and it does not have effect on reliability of the provided results.>>

**Rev1_5**: The variant inclusion and exclusion data is unclear. For example, having an exclusionary criteria which appears to be <100 individuals is not appropriate as coverage in gnomAD is variable. Thus, a poorly covered area with fewer individuals sequenced may thus have fewer minor allele positive individuals that are nonetheless a high minor allele frequency. Minor allele frequency should be used.

Inclusion criteria for missense variants are particularly unclear. What criteria is used to determine whether a missense variant is included?

RESPONSE: <<The criteria is well defined in the manuscript. Changing it would not have any significant effect on the outcome even though it would be more orthodox >>.

**Rev1_6**: Table 1 seems to be extraneous and seems odd. I think it should be removed and potentially referenced in the Discussion

RESPONSE: << We think that table 1 summarizes well what is known about clinical associations of biallelic truncating NRAP variants. Other reviewers did not see it odd, so we feel that it can be present in the introduction or discussion section. If it is considered mandatory, we are ready to move it into the discussion section.>>

**Rev1_7:** What was the pathogenicity assessment of the NRAP variants by 2015 ACMG guidelines?

RESPONSE: << Yes, ACMG criteria was used. >>

**Reviewer 2**

Koskenvuo et al. presented interesting novel data on NRAP gene’s implication in the pathogenesis of autosomal recessive dilated cardiomyopathy (DCM).
Authors reviewed genetic results of 577 patients either with a confirmed or suspected diagnosis of DCM and compared them with data of control cohort of 31062 individuals, including both non-cardiac controls (n>25000) and non DCM cardiac controls (n>5000).
They identified two rare variants in NRAP gene [(four homozygous truncating (PTV), or compound heterozygous (one patient had two different PTV and 6 had PTV/missense)] in 11 unrelated probands with DCM (1.9%) but none in the controls, with OR of 1052 in comparison to controls. They concluded that NRAP variants could explain 0.25%-2.46% of all DCM cases.
Also, Authors stress the fact that NRAP patients seem to have an earlier onset of major cardiac end-points compared to cardiac laminopathy or DCM, and higher requirement for cardiac transplantation/LVAD.
This is a very important, well designed and rigorously performed study, rather nicely presented.

**Rev2_1**: However, page 10: Authors state: “In these 11 individuals four had a homozygous PTV, one had two heterozygous PTVs and two were compound heterozygous for a PTV/missense variant.” This adds up to 7 , and not to 11.
Although later we can learn that the same variants were present in other patients, and the Table 2 lists them, it is unclear and should be corrected.

RESPONSE: << We have modified the table 2 to present homozygous/compound heterozygous mutation status in more understandable way >>.

**Rev2_2**: Page 19, Authors state:
"In the first study suggesting association of NRAP with cardiomyopathy, the proband’s 35-year-old brother who was compound heterozygous for two PTVs in NRAP”. This is untrue. As Authors correctly state in the Table 1, the first identified variant was homozygous nonsense NRAP variant (p.Arg1502*).

RESPONSE: << Corrected (compound heterozygous to homozygous) >>.

Reviewer #3: Koskenvuo et al report a study where they identified 4 cases with biallelic NRAP protein truncating variants (PTV), 2 cases with NRAP compound heterozygous PTV and a missense variant, 1 case with two PTVs (phase unknown) and 4 cases with a PTV and a missense variant (phase unknown), in a large dilated cardiomyopathy cohort (n=577). The aim was to assess the association of bi-allelic loss of function (LoF) variants in NRAP with autosomal recessive cardiomyopathy, since previously, only 4 such cases have been reported. Large disease cohorts are a useful resource to assess for the presence of any gene’s variants and to study their association with the disease. This study provides supports the role of NRAP biallelic loss of function variants and their association with autosomal recessive cardiomyopathy, though with reduced penetrance. The data presented in the study does not support for a causal role of NRAP LoF variants in autosomal dominant dilated cardiomyopathy. However, this manuscript in its current form needs substantial revision before it is suitable for publication in PLos One. This report can benefit from addition of phenotype details of the affected individuals and further discussion on incomplete penetrance or age related penetrance etc.


Major comments:

**Rev3_1**: In this study the authors found 6 cases with biallelic NRAP variants (4 homozygous & 2 with PTV and a missense variant in trans/compound heterozygous). This needs to be clearly stated in the text as the other 5 sets of NRAP variants may or may not be in trans.

RESPONSE: << Fixed; In these 11 individuals, four had a homozygous PTV, one had two heterozygous PTVs (phase unknown) and two were compound heterozygous for a PTV and a missense variant (*in trans*). In five patients, the phase of the *NRAP* variants was unknown. >>.

**Rev3_2**: Were the variants Sanger confirmed in the 11 cases ? If so, please mention that in the methods.

RESPONSE: << No, they were not. We follow a validation and related variant allocation workflow of >5000 samples with NGS and Sanger data, which yield 100% specificity for a variant identified by NGS when certain QC criteria are passed. All the identified *NRAP* variants fulfilled all criteria to be true positive call, and they were manually inspected using IGV software.>>

**Rev3_3**: Table 1
• It is best to list the variants in the “chr-genomic coordinate- Ref allele- Alt allele” format. This format is most useful along with the HGVS nomenclature for the cDNA , protein and the coding exon to which the LoF variants maps to (for example 3/20).

RESPONSE: << Table 1 is fixed as requested. >>

**Rev3_4**: Table 1
• Please add the units and reference ranges for the CK & ProBNP levels listed

RESPONSE: << All available data added. >>

**Rev3_5**: Table 1

• Addition of the gender of the affected individual will also be helpful

RESPONSE: <<Added, please see table 1>>.

**Rev3_6**: Table 1

• In the footnotes, it needs to be mentioned that homozygotes for these variants in Gnomad were not seen.

RESPONSE: <<Added, please see table 1 >>

**Rev3_7**: Table 1

• Please mention the NRAP NM_ID used by the published reports listed in this table.

RESPONSE: << Added, please see table 1 >>.

Table 2
• Table 2 contains information on the cases reported in the current study; however, the organization of this table is not reader friendly.

**Rev3_8**: Table 2
• Please reword the legend for clarity and make it concise
Suggested Legend “NRAP variants identified in individual with confirmed or suspected dilated cardiomyopathy”

RESPONSE: << We modified it to be even more concise>>.

**Rev3_9**: Table 2• It is best to have one row each for one affected individual

RESPONSE: <<We agree. We modified the table 2 so that it is easy to follow as patient number is at left and the patient’s variants are listed within one row if homozygous and in two rows if compound heterozygous or had two variants (phase unknown) >>.

**Rev3_10**: Table 2
• Please add available phenotype information for each individual including gender, age of onset, CK levels, available cardiac biopsy

RESPONSE: << All available data is now included. >>.

**Rev3_11**: Table 2
• It is best to list the variants in the “chr-genomic coordinate- Ref allele- Alt allele” format

RESPONSE: <<We modified the table and decided to use HGVS nomenclature. >>

**Rev3_12**: Table 2

List the variants in Hgvs nomenclature (cDNA and protein)

RESPONSE: << Please see above. >>

**Rev3_13**: Table 2
• Add a column to clarify whether it is compound heterozygous or phase unknown

RESPONSE: << Homozygotes and compound heterozygous patients are now marked with bold font >>.

**Rev3_14**:

• The cases where unambiguous biallelic NRAP variants were seen (6/11) should be listed first

RESPONSE: << Changing the cases numbers or the order may add confusion at this stage, thus not done.>>

**Rev3_15**:
• the number of coding exon where the variant is located will be a useful addition especially, for the C terminal LoF variants, to assess whether they are predicted to undergo NMD or will result in truncation

RESPONSE: << Done >>.

**Rev3_16**:
• The “type of variant” column is redundant and may be removed

RESPONSE: << We decided to left this info as it may be helpful for those not expert in genetics. >>

**Rev3_17**:
• SIFT and conservation data can be listed in the footnotes

RESPONSE: << The table is modified to be more concise. >>

**Rev3_18**:
• In the footnotes, it needs to be mentioned that homozygotes for these variants in Gnomad were not seen

RESPONSE: << Done >>.

**Rev3_19**: For calculation of the population based prevalence using the frequency of NRAP LoF variants in Gnomad v.2.1, were the NRAP LoF variants seen in the last coding exon also included? The LOF variants in the last coding exon of NRAP may not undergo NMD and may result in a truncated product. Hence if these were included, the prevalence estimate derived for NRAP associated recessive cardiomyopathy might be a slight overestimate. Further, there were two high quality homozygous LoFs seen for NRAP in gnomad v.2.1.1 – were they included/removed for the prevalence calculation?

RESPONSE: << None of the LoF variants detected in our DCM cohort locate within the last or penultimate exon, thus they are not expected to escape from NMD. The used reference set consist all LoF variants, none removed, which is orthodox way to calculate odds ratio (OR)/ enrichment. If we would remove the C-terminal truncations, the OR would actually increase. Thus, our approach does not lead to overestimation of the effect. >>.

**Rev3_20:** Discussion
If the age of onset data is available for the 6 individuals, along with the 4 published reports the heterogeneity seen should be discussed. If a large variability is seen, it might be supportive of age related penetrance. In addition, the age of onset or its variability, if seen for other AR non syndromic dilated cardiomyopathy genes, is also worth a mention.
Since NRAP is predominantly expressed in the heart & skeletal muscle, in the 6 cases with bialleleic variants were there any muscle issues seen in the affected individuals? Were any muscle issues noted in the 4 previously published cases? If so, this needs to be mentioned in the discussion

RESPONSE: <<The age of onset is added if available for all cases; both those described in literature and those reported in the manuscript. There is substantial variability on age of onset and it seems that two LoF variants lead earlier presentation compared to LoF + missense. In general, there is substantial variability on penetrance and age of onset in all dominant and recessive cardiomyopathies and the reasons are poorly understood at the moment. None of the DCM patients with biallelic or potentially biallelic *NRAP* variants in our study had known skeletal muscle involvement, and this information is added to footnotes of Table 4. Moreover, historical cases have neither and we added CK values for those cases when available (Table 1) >>.

**Rev3_21:** The authors list a handful of genes, which cause AR non-syndromic cardiomyopathy. Is age related penetrance or incomplete penetrance a feature seen for these genes as well ? in individuals who carry biallelic variants in these genes. Or this is uniquely seen for NRAP?
This will be insightful; Further, Gnomad V.2.1.1 data has two high quality homozygous LoF variants in NRAP. The authors should attempt to provide a potential explanation for this observation.

It is interesting that a single missense variant (p.Gln24His) which could potentially affect splicing is seen in 4 affected individuals.

RESPONSE: <<Please see above, response Rev3_20. It is not surprising that there a few, actually three homozygous LoF variants in gnomAD. However, one of them (<https://gnomad.broadinstitute.org/variant/10-115412782-T-TCAATCACCTGTAATAAATTTAAAAATGGCTTATAATTG?dataset=gnomad_r2_1>) is is likely a false positive call, the second (<https://gnomad.broadinstitute.org/variant/10-115412781-ATAGTCTTCTGTATATTCCTGTTGGTCAGAACCAATGTCAGCATGAGAACTGAGATGATATTGAATGACAACTAAGAAAACCGCAATAAAACCAATGAAACTAGATTCATAAAGTTCTTCGGAAATATT-A?dataset=gnomad_r2_1>) may not lead to loss-of-function as it is not predicted to cause absent protein by Alamut, which predict a rescue/cryptic in-frame splice site and the third (<https://gnomad.broadinstitute.org/variant/10-115349424-C-T?dataset=gnomad_r2_1>) affects the last natural splice donor site (exon 41/intron 41 boundary), and is thus expected to escape from NMD. Moreover, there are many individuals with well established cardiomyopathy mutations in reference population databases as many patients get affected at old age and may thus be selected as healthy individuals to the reference set. >>

Minor comments
Several sentences lack clarity and I urge the authors to read through the manuscript carefully and edit the manuscript for clarity and typos.
For example,

**Rev3_22:** Title
“ Biallelic loss-of-function in NRAP is a common cause of recessive dilated cardiomyopathy”
Instead the following title is clearer
“ Biallelic loss-of-function variants in NRAP are a common cause of recessive dilated cardiomyopathy”

RESPONSE: << We think that the original title is better as missense variants may not be considered as loss-of-function variants, and thus the proposed title has also a conflict when considering the findings of the work>>.

Abstract
**Rev3_23**: “Confirmed or potentially biallelic NRAP variants were enriched in DCM”
the usage of the word confirmed is confusing ; instead it is suggested to use “Biallelic or potential bi-allelic”

RESPONSE: << Done >>.

**Rev3_24:** “Biallelic (n=6) or two (n=5) NRAP variants (two PTVs or PTV+missense) were”
(two PTVs or PTV+missense)
Instead the following addition will make it more clear.
“Biallelic (n=6) or two (n=5) NRAP variants (two PTVs or PTV+missense) were”
(two PTVs or PTV+missense, phase unknown )

RESPONSE: << Done >>.

Methods: Patients

**Rev3_25:** Please clearly mention whether the HQSA also included NRAP.

RESPONSE: << Yes, included>>.

**Rev3_26:** Page 5
estimated int the range of 1:500 to 1:3,000 [2–4].

RESPONSE: << Done >>.

**Rev3_27:**Page 10:

“Visual Splicing software v2.11 (Interactive Biosoftware, France) predicts that this variant either leads to either loss of the native splice donor”

Instead

“Visual Splicing software v2.11 (Interactive Biosoftware, France) predicts that this variant either leads to loss of the native splice donor”

RESPONSE: << Done >>.

**Rev3_28:** “Enrichment of NRAP variants in DCM
We identified two rare NRAP variants, of which at least one was a PTV in 11 out”
Instead
We identified cases, which carried two rare NRAP variants, of which atleast one was a PTV

RESPONSE: << Done >>.

**Rev3_29:** Gene names should be italicized in the manuscript text.

RESPONSE: << Gene names are in italics unless when used in context to the same protein.>>

Reviewer #4: The authors identified biallelic loss-of-function in NARP as a common cause of recessive dilated cardiomyopathy with next generation sequencing. However, there are some issues to address.
Major issues:
**Rev4_1**: The study didn’t detect the variants of well-established causal genes of DCM. Is the DCM phenotype caused by biallelic loss-of-function in NARP or variants of the known causal genes, such as TTN, RBM20, et, al? It would better to perform whole exome sequencing or targeted next generation sequencing with causal genes of DCM on the cohort.

RESPONSE: <<We did see variants in the other well-established cardiomyopathy genes but they are out of the scope of this work. Importantly, none of the 11 patients with two *NRAP* variants had an alternative molecular diagnosis identified in either large NGS panel (n=9) or exome sequencing (n=2). This is mentioned in the results section.>>

**Rev4_2**. It would be better to confirm the variants in NARP by Sanger sequencing, especially in family segregation analysis due to the false positive rate of next generation sequencing.
Minor issues:

RESPONSE: <<The NGS results were achieved using ISO-15189 and CAP accredited NGS pipeline validated using >5000 samples with NGS and Sanger data, which yield 100.00% specificity for a variant identified by NGS when certain QC criteria are passed. All the identified *NRAP* variants fulfilled all QC criteria to be true positive call.>>

**Rev4_3.** Vasilescu et, al reported NRAP underlying childhood dilated cardiomyopathy. It would be better to mention their work in discussion section. (J Am Coll Cardiol. 2018 Nov 6;72(19):2324-2338.) in NARP.

RESPONSE: << Done >>.
